# A novel approach to enhance beta-sitosterol bioavailability isolated from *Jurinea macrocephala* (a high-altitude) plant in biodegradable chitosan against lung cancer

**DOI:** 10.3389/fonc.2025.1659465

**Published:** 2025-12-08

**Authors:** Monica Sangral, Ankita Singh, Ashutosh Gupta, Madhulika Bhagat

**Affiliations:** 1 School of Biotechnology, University of Jammu, Jammu, India; 2 Radiation and Oncology Division, Government Medical College, Jammu, India

**Keywords:** *Jurinea macrocephala* (a high altitude) plant, β-sitosterol, chitosan, lung cancer, apoptosis

## Abstract

**Introduction:**

Lung cancer remains a significant health challenge, often resulting in a poor prognosis due to the limited availability of effective targeted therapies. Beta-sitosterol (BS), extracted from *Jurinea macrocephala*, possesses diverse pharmacological properties but faces limitations such as poor solubility and low bioavailability. This study aims to address these limitations by encapsulating β-sitosterol in chitosan nanoparticles, thereby enhancing its solubility and controlled and sustained release to improve therapeutic efficacy against lung cancer with an emphasis on exploring its potential mode of cell death.

**Methods:**

BS was isolated using column chromatography, with high-performance liquid chromatography (HPLC) confirming its purity. The ionic crosslinking method with tripolyphosphate (TPP) was employed to formulate chitosan-based nano beta-sitosterol (BSC). The characterization of the nanoparticles was performed using field emission scanning electron microscopy (FE-SEM), dynamic light scattering (DLS), X-ray diffraction (XRD), and Fourier transform infrared spectroscopy (FTIR). Cell-based assays, including 4′,6-diamidino-2-phenylindole (DAPI) staining, colony formation, apoptosis, and cell cycle analysis, were conducted to assess the effects of nano β-sitosterol compared to its pure form on A549 lung cancer cell lines.

**Results:**

β-Sitosterol (0.6% w/w) was isolated from *J. macrocephala* chloroform extract collected from the Bhaderwah region of Jammu and Kashmir. The resulting nanoparticles had an average size of 190 nm, a polydispersity index of 0.592, and a zeta potential of 16.3 mV. The nanoparticles displayed encapsulation efficiency of 76% ± 2.19% with a loading capacity of 3.04 ± 2.08. *In vitro* release studies have demonstrated a sustained release of β-sitosterol over 7 days. Cell-based assays indicated that nano β-sitosterol had a more prolonged and controlled effect on cell proliferation, nuclear distortion, cell cycle progression, and apoptosis in A549 cells compared to free β-sitosterol. This study is the first to compare the effects of nano β-sitosterol versus pure β-sitosterol on apoptosis and cell cycle progression in a time-dependent manner.

**Discussion:**

Chitosan-encapsulated nano β-sitosterol demonstrated enhanced solubility, sustained release, and improved therapeutic efficacy against lung cancer, highlighting its potential as a promising delivery system for lung cancer with improved therapeutic outcomes.

## Introduction

Lung cancer is a significant public health concern, causing a considerable number of deaths globally, with GLOBOCAN 2020 reporting 1.8 million deaths (18%) in 2020. According to the American Cancer Society, in 2024, there were approximately 234,580 new cases of lung cancer (116,310 in men and 118,270 in women) in the USA. Among them, 125,070 deaths from lung cancer (65,790 in men and 59,280 in women) were reported (1). Current treatments for lung cancer primarily involve radiation therapy, surgery, and chemotherapy. However, these approaches face significant limitations, including drug resistance, lack of specificity, poor selectivity, high dosage demands, low bioavailability, and severe side effects, which reduce their overall efficacy (2, 3). These challenges have prompted a shift toward exploring safer and more effective chemotherapeutic alternatives. Natural products, particularly phytochemicals from plants, have emerged as promising candidates for anticancer therapy, with approximately 75% of anticancer drugs in use derived from natural sources (4). The advantages of natural compounds include fewer adverse effects and their ability to influence multiple downstream pathways involved in cancer progression. This may explain why, among the 240 anticancer drugs approved in the past four decades, only 29 are exclusively synthetic. Furthermore, over the last decade, several synthetic compounds that mimic the pharmacophores of natural products have been approved as antitumor agents (5, 6). Plant-based drugs have been extensively explored for cancer chemotherapy, with approximately 70% undergoing clinical trials at various stages (7). Consequently, while natural compounds exhibit promising therapeutic properties, they face several challenges that need to be addressed, including issues with solubility, stability, rapid clearance rates, and biocompatibility. Recently, the integration of nanotechnology into drug delivery systems for cancer treatment and diagnosis has garnered considerable attention in preclinical research. Nanomedicine integrates concepts from pharmaceutical sciences and nanoscience, employing organic and inorganic nanoparticles (NPs) with polymeric structures at the nanoscale (8). The development of these structures can enhance drug delivery effectiveness by improving the therapeutic index, facilitating targeted delivery, and allowing for controlled drug release. Furthermore, nanotechnology has the potential to enhance various pharmaceutical properties of drugs, such as their stability, solubility, and circulation half-life (9, 10). Currently, several nanomedicines have received approval for therapeutic use. For instance, liposomal doxorubicin is employed in treating Kaposi’s sarcoma, offering enhanced delivery to tumor sites while reducing systemic toxicity (11). Leuprolide acetate combined with the polymer poly(dl-lactide-*co*-glycoside) (PLGA) is utilized for prostate cancer, providing extended circulation times. Furthermore, albumin-bound paclitaxel has demonstrated improved solubility and delivery for breast and pancreatic cancers (12). Chitosan, a chitin-derived natural polysaccharide, has garnered significant consideration as a nano-vehicle due to its variable properties such as biodegradability, biocompatibility, hydrophilicity, and non-toxicity (13). As the only naturally occurring cationic polymer, chitosan imparts exceptional physical and biochemical attributes, including enhanced permeability, transfection efficiency, mucoadhesion, flexibility, and the ability to encapsulate hydrophobic drugs (14). These characteristics make chitosan a versatile and cost-effective carrier, particularly for drug delivery systems targeting epithelial tissues. Chitosan nanoparticles (CSNPs) provide additional benefits by protecting drugs from external factors like pH and enzymatic degradation while promoting sustainable release, ultimately improving drug bioavailability [15.16]. These properties position chitosan as a promising ingredient for developing progressive drug delivery systems, particularly in the field of chemotherapeutic drugs (15). The present study aimed to isolate potential anticancer phytoconstituents from *Jurinea macrocephala* root extracts and to formulate tripolyphosphate (TPP)-crosslinked chitosan nanoparticles using the ionic gelation method. The particle size, shape, structure, stability, percent encapsulation, and drug release profile of the molecule from the chitosan nanoparticles were thoroughly characterized using various analytical techniques. Additionally, the study evaluated the anticancer efficacy of chitosan nanoformulation compared to the free molecule, focusing on its effects against lung cancer, specifically A549 cells.

## Materials and methods

### Plant collection, extraction, and fractionation

*J. macrocephala* Benth. roots were collected in a sterilized bag from the Bhaderwah district of Jammu and Kashmir (32°59′N, 75°43′E). Experts from the Department of Botany, University of Jammu, authenticated the plant, and a specimen voucher number (HBJU16000) was assigned. The roots were shaded, dried, powdered, and macerated in chloroform for 24 hours. The filtrate was then enriched using a rotary evaporator (R-200, Buchi Rotavapor, Eastern Switzerland) (16). The remaining plant residue was fractionated sequentially, repeating the above process using ethyl acetate, acetone, methanol, and water. All prepared fractions were stored at −20°C. The fractions collected were named as chloroform (JMC), ethyl acetate (JMEA), acetone (JMAC), methanol (JMM), and aqueous (JMW).

### Isolation of beta-sitosterol using column chromatography

The chloroform extract of *J. macrocephala* (25 g) was fractionated through a column packed with normal phase silica gel (60–120 mesh) using ethyl acetate:hexane (1% to 10% v/v) as a solvent system (17). Elution resulted in 100 fractions and was labeled as 1JM to 100JM. The collected fractions remained undisturbed until the evaporation of the solvents to obtain the isolated compound. From the fractions of *J. macrocephala* chloroform root extract, fraction 29JM was selected and subjected to physical and chemical characterization.

### Characterization of beta-sitosterol molecule

High-performance liquid chromatography (HPLC) was used to identify fraction 29JM. The analysis was carried out using HPLC-DAD equipment (Agilent, Waldbronn, Germany) using a C18 analytical column (5-μm particle size, 4.6 × 250 mm) (18). Thin-layer chromatography (TLC) analysis for the identification of fraction 29JM was carried out. For this method, different mobile phases and detection reagents were developed (19). One-dimensional NMR (^1^H, ^13^C, and DEPT-135) techniques were performed to elucidate the structure of the isolated compound (20). Resonance spectrum was performed using the instrument Bruker, Rheinstetten, Germany (AMX 400 MHz) with a standard sequence of pulse, operational at 400 and 100 MHz for ^1^H and ^13^C NMR, respectively (21). Chemical shifts (parts per million) were established with respect to the chemical shifts of the solvent, i.e., deuterated chloroform (CDCl_3_) and Dimethyl sulfoxide (DMSO), obtained. Coupling constants (J-values) were recorded in hertz (Hz).

### Preparation of drug-loaded nanoparticles

Initially, 1% (w/v) chitosan polymer was dissolved in 2.0% (v/v) aqueous acetic acid to prepare a homogeneous chitosan solution (0.15% w/v) (22). Different ratios of molecule and chitosan solution (1:1, 1:2, 1:4, and 1:8) were stirred with dropwise addition of TPP (0.1% w/v). After 4 hours of continuous stirring, the solution was centrifuged to collect the pellet, which was further dried and lyophilized.

### Characterization of chitosan-encapsulated beta-sitosterol

The chitosan nanoparticles were further characterized to ensure the success of encapsulation by various techniques (23). Scanning electron microscopy was employed to analyze the visual morphology of nano-encapsulations (JSM6100, Jeol, Tokyo, Japan). The size (diameter), particle size distribution, and stability of chitosan encapsulations were examined using a digital light scattering analyzer (MAL105726, Malvern, Worcestershire, UK). Zeta potential was measured using a laser Doppler electrophoresis module in a dynamic light scattering (DLS) analyzer. Measurements were conducted at 25 °C ± 0.1°C in disposable folded capillary cells. Each sample was equilibrated for 2 minutes before measurement. The Smoluchowski approximation method was applied to calculate the zeta potential from the electrophoretic mobility. For each condition, three independent measurements were taken and averaged to ensure reproducibility. X-ray diffraction (XRD) was performed to investigate the structure of the chitosan nanoparticles using an X-ray diffractometer (Power-based). Various functional groups in the nanoparticles were analyzed using Fourier transform infrared spectroscopy (FTIR) (RZX, PerkinElmer, Shelton, US).

### Encapsulation efficiency and loading capacity

The encapsulation efficiency (the amount of biomolecule successfully captured inside a nanoparticle) and loading capacity (drug loaded on nanocomposite) of chitosan nano β-sitosterol were determined using a UV spectrophotometer (24). The amount of biomolecule in the suspension was analyzed by centrifuging nanoparticles at 1,000 rpm for 30 minutes and measuring the concentration of the drug in the supernatant at 425 nm. The concentration of the biomolecule was analyzed after necessary dilutions. The amount of β-sitosterol entrapped in the nanocomposite was determined using the equations given below.


Encapsulation efficiency (EE%)= [Total  amount of β-sitosterol  added −non-encapsulated β-sitosterol/Total amount of β-sitosterol]×100

Loading capacity (LC%)= [(Total amount of drug −Free amount of drug)/Nanoparticle weight]×100

### *In vitro* release assessment of biomolecules

After the characterization of chitosan nanoparticles, their release profiles were established using a dialysis membrane (25). The release profiles of β-sitosterol and nano β-sitosterol were performed under buffered conditions using Phosphate buffered saline (PBS) at pH 7 at room temperature. Beta-sitosterol (1 mg/mL) was weighed precisely, placed in properly sealed dialysis bags (Sigma, Darmstadt, Germany, D9277), and dispersed in PBS. PBS was extracted from each sample at different time intervals (up to 4 hours and further up to 7 days). The absorbance of the extracted sample was recorded to calculate the concentration of the released molecule using the standard curve equation of the pure molecule. Absorbance was determined for beta-sitosterol at 240 nm.

### Cell culture and cytotoxicity assay

Human lung (A549) cells were cultured in Dulbecco’s modified Eagle’s medium supplemented with 10% fetal bovine serum at 5% CO_2_ and 37°C. Briefly, cells were seeded at a density of 1 × 10^3^ cells per well in a 96-well plate. Different concentrations (1, 5, 10, and 20 μg/mL) of the sample were added to each group (triplicate wells) and incubated for 24 hours. Ten microliters of MTT solution (5 mg/mL) was added to each well, and absorbance was recorded at 570 nm using a multimode reader (Tecan’s , Mannedorf, Switzerland Infinite^®^ F200). The inhibitory concentration (IC_50_) values were calculated relative to the control (6).

### Colony formation assay

Lung A549 cells (10^3^ cells/well) were seeded in a 6-well plate (Tarson, West Bengal, India, 980010). Cells were treated with 11 μg/mL of beta-sitosterol (BS) and chitosan-based nano beta-sitosterol (BSC). The treatment was given over 72 hours, followed by washing of cells using PBS and cell fixation using 70% methanol. Next, staining of cells with crystal violet (0.5%) was performed, and stained cells were placed in the dark for 15 minutes. After 15 minutes, the stain was decanted, followed by washing of cells using PBS. Visualization of cells using fluorescence microscopy was further carried out at ×20 (FLoid Cell Imaging Station, Life Technologies, Bothell, WA). Colonies per well were counted, and the number of colonies was expressed as an average of colonies in five fields (keeping the number of fields, n = 5).

### Nuclear staining

In order to study the mode of cell death, a nuclear staining technique using nuclear dye, 4′,6-diamidino-2-phenylindole (DAPI), was performed (12). A549 cells (1 × 10^3^ cells/well) were seeded in a 6-well plate. Cells were treated with 8 μg/mL of β-sitosterol and nano β-sitosterol. The treatment was given over 72 hours, followed by washing of cells using PBS. Next, cells were stained with DAPI and incubated for 15 minutes in the dark. After 15 minutes, the stain was decanted, followed by washing of cells using PBS. Visualization of cells using fluorescence microscopy was further carried out at ×20 (FLoid Cell Imaging Station, Life Technologies). DAPI-stained nuclei per well were visualized, and their morphology was examined further by counting the number of distorted nuclei per field (keeping the number of fields, n = 5).

### Cell apoptosis study

A density of 10^3^ cells/well was used for seeding of A549 cells in a 6-well plate. Cells were treated with 11 μg/mL of β-sitosterol and nano β-sitosterol. The treatment was given in two time sets, i.e., 48 and 72 hours. After 48 and 72 hours of treatments, cells were trypsinized and centrifuged, and the pellet was then washed with ice-chilled PBS. Pellet was suspended in a 1× binding buffer with subsequent staining with 0.25 μg/mL Annexin V and 0.25 μg/mL propidium iodide. Stained cells were placed in the dark for 15 minutes. The percentages of cells in early or late apoptosis were examined using Flouoscent activated cell sorting (FACS) (FACS Accuri, C6 Plus, furnished with 488-nm excitation laser). Camptothecin (1 μM) was used as a positive control (26).

### DNA cell cycle analysis

A density of 10^3^ cells/well was used for seeding of A549 cells in a 6-well plate. Cells were treated with 11 μg/mL of β-sitosterol and nano β-sitosterol. The treatment was given in two time sets, i.e., 48 and 72 hours. After 48 and 72 hours of treatments, cells were trypsinized and centrifuged, and the pellet was then washed with ice-chilled PBS. Following 70% ethanol fixation, staining of cells with propidium iodide (25 μg/mL) was performed, and stained cells were placed in the dark for 15 minutes. The proportions of cells in various cell cycle stages were examined using FACS (Accuri™, C6 Plus) (27).

### Molecular docking of β-sitosterol against anti-apoptotic protein NRF1

To study the interaction of β-sitosterol with anti-apoptotic protein through molecular docking, the protein and ligand molecules were imported into Molegro Virtual Docker and visualized for drug interaction sites. The homologous protein sequences for nuclear respiratory factor 1 (NRF1) were retrieved from the NCBI database, and the structure of the protein molecule was constructed using the Swiss-Prot homology modeling software. The protein structure thus obtained was then validated and visualized using the PyMOL visualization tool. The structure of ligand molecules was retrieved from the ZINC database in sdf format. The drug was then analyzed using the Lipinski filter. The Molegro Virtual Docker and AutoDock were used for protein–molecule interaction.

### Statistical analysis

All analyses were performed using GraphPad Prism Ver. 6.0. Data were expressed as mean ± SEM (n = 3) with statistical significance indicated when p < 0.05.

## Results

### Isolation of potential phytoconstituents from *J. macrocephala* chloroform extract

**Figure 1:**
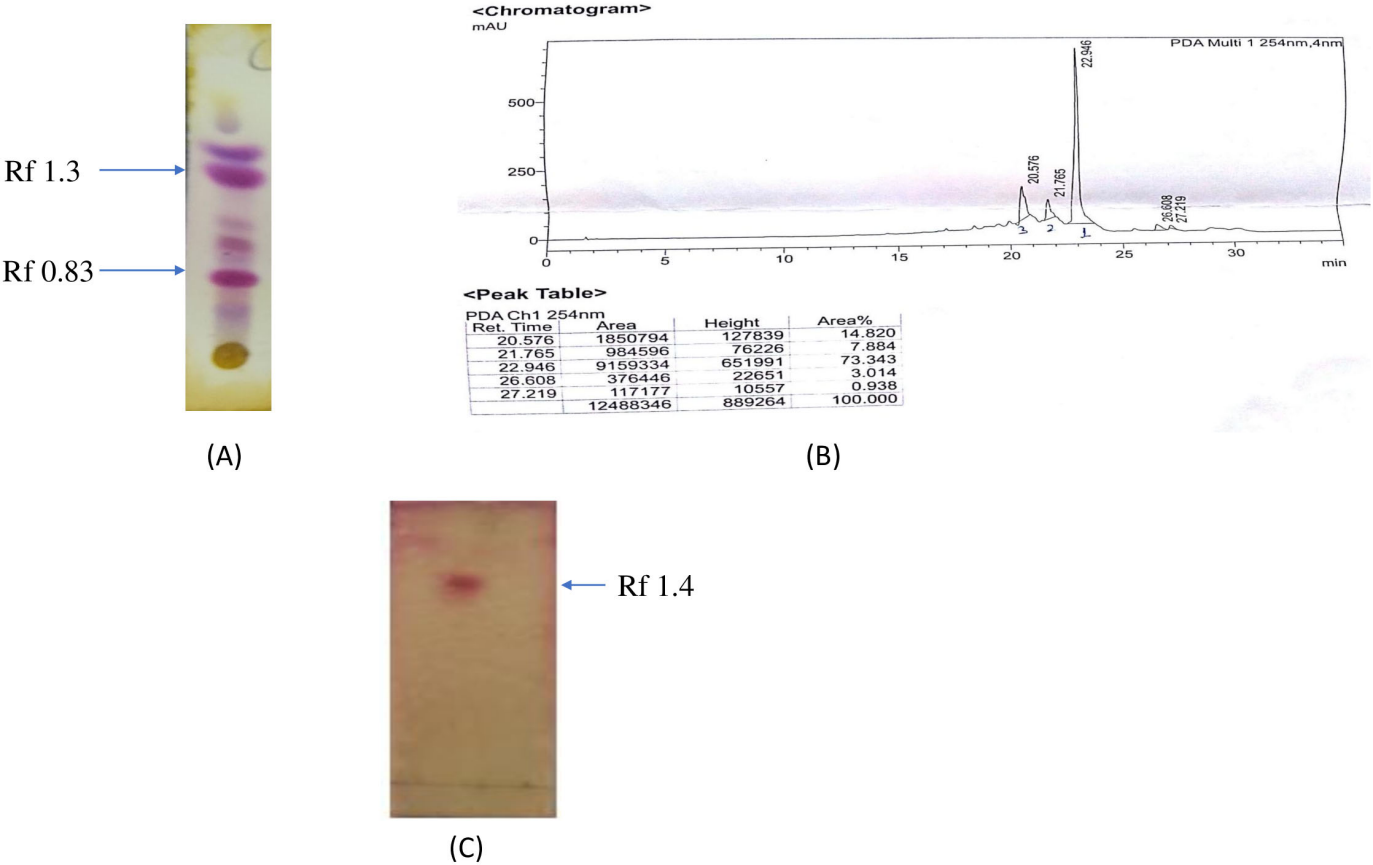
Characterization of fraction 29JM. **(A)** TLC strip of *Jurinea macrocephala* chloroform root extract depicting two major spots of retention factor of 0.83 and 1.3. **(B)** HPLC chromatogram of fraction 29JM, revealing one major peak at retention time of 22.946 with 73.343 area percentage. **(C)** TLC of subfraction 29JM displaying single spot. TLC, thin-layer chromatography; HPLC, high-performance liquid chromatography.

The column chromatography technique was used to achieve the objective. Precisely weighed (25 g) chloroform extract was placed on the column silica bed (60–120 mesh) and eluted with increasing concentrations of solvent mixtures, i.e., starting from 1% v/v (ethyl acetate:hexane) to 10% v/v (ethyl acetate:hexane). The eluted mixture of chloroform (JMC) extract with 1% to 10% of ethyl acetate in hexane yielded 100 fractions and was labeled (1JM to 100JM). From all the eluted and labeled fractions, fraction number 29JM, which was eluted with a solvent mixture of 4% ethyl acetate in hexane, obtained as a white powder (0.31 g, 1.24% w/w), was further selected for analysis via TLC (**Figure 1A**) and HPLC. Fraction 29JM showed three peaks on HPLC chromatograms, with the prominent one at a retention time of 22.946 with 73% area (**Figure 1B**). Fraction 29JM was then subjected to sub-fractionation through column chromatography for the elution of pure molecule using the mixture of 4% to 5% ethyl acetate in hexane. Furthermore, subfraction 29JM obtained as white powder (0.15 g, 0.6% w/w), giving a single spot on TLC (**Figure 1C**), was subjected to NMR analysis for its characterization.

### Characterization of subfraction 29JM

**Figure 2:**
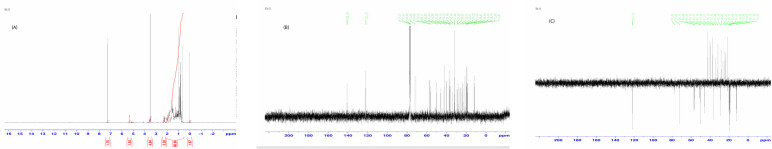
**(A)**^1^H NMR of subfraction 29JM. **(B)**^13^C NMR of subfraction 29JM. **(C)** DEPT-135 NMR of subfraction 29JM.

While proceeding with subfraction 29JM for its characterization, NMR was chosen as a standard approach for the identification of the molecule. Therefore, ^13^C NMR, ^1^H NMR, and DEPT-135 NMR were performed for subfraction 29JM. ^13^C NMR determines the information about the nature of carbon atoms present in the molecule, ^1^H NMR determines the position of hydrogen atoms, and DEPT-135 determines the multiplicity of carbon atoms (**Table 1**). The ^1^H NMR spectrum demonstrated the presence of 50 hydrogens: six hydrogens as CH_3_, 11 hydrogens as CH_2_, nine hydrogens as CH, and one hydrogen as an OH group. The appearance of the singlets at δ 1.03 and 0.70 was corroborative of the presence of two molecules of CH_3_ attached to quaternary carbons. Complex multiplets at δ 2.32 and 2.04 revealed that the two molecules of CH_2_ were adjacent to the carbon attached to the OH group. The multiplet at δ 3.54 was due to a proton connected to the carbon that was attached to an OH group (**Figure 2A**). The ^13^C NMR spectrum showed the existence of 29 carbons [CH_3_, CH_2_, CH, and quaternary carbon (QC)] (**Figure 2B**). DEPT-135 spectrum indicated the presence of 26 carbons: six peaks up due to the presence of CH_3_ groups, nine peaks up for CH groups, and peaks down due to the presence of 11 CH_2_ groups (**Figure 2C**). According to the results of the three NMR spectra (^1^H NMR, ^13^C NMR, and DEPT-135), the compound was identified as beta-sitosterol ^12^. Hence, various characterization approaches confirmed that the isolated subfraction 29JM from the chloroform root extract of *J. macrocephala* Benth. contained β-sitosterol. Here, the β-sitosterol molecule was obtained with 0.6% w/w. Furthermore, the physical properties of subfraction 29JM were also analyzed and correlated with those of beta-sitosterol. Subfraction 29JM appeared as a white-colored waxy powder, was insoluble in water and soluble in ethanol, and had a melting point of 140°C, determined using the capillary method. The physical properties of subfraction 29JM analyzed were in accordance with the physical characteristics of compound β-sitosterol (white waxy powder, soluble in alcohols, and melting point in the range of 139°C to 142°C) (16).

**Table 1:** Chemical shift (ppm) values of ^1^H NMR and ^13^C NMR for subfraction 29JM.

S.N no	C and H atoms	Chemical shift (ppm)	
		^13^C NMR	^1^H NMR
1	CH_2_	37.28	1.46 (m)
2	CH_2_	31.69	1.56 (m)
3	CH(CH)	71.82	3.54 (m)
4	CH_2_	42.33	2.32 (m)
5	QC (=)	140.77	–
6	CH (=)	121.73	5.37
7	CH_2_	31.93	2.04 (m)
8	CH	31.93	1.69 (m)
9	CH	50.16	1.55 (m)
10	QC	36.51	–
11	CH_2_	21.11	1.52 (m)
12	CH_2_	39.80	1.51 (m)
13	QC	42.34	–
14	CH	56.79	1.50 (m)
15	CH_2_	24.33	1.58 (m)
16	CH_2_	28.27	1.85 (m)
17	CH	56.08	1.45 (m)
18	CH_3_	11.89	0.70 (s)
19	CH_3_	19.42	1.03 (s)
20	CH	36.17	1.60 (m)
21	CH_3_	18.84	0.94
22	CH_2_	33.98	0.93 (m)
23	CH_2_	26.11	1.15 (m)
24	CH	45.86	1.38 (m)
25	CH	29.19	1.57 (m)
26	CH_3_	19.84	0.84
27	CH_3_	19.06	0.86 (d)
28	CH_2_	23.10	1.10 (m)
29	CH_3_	12.01	0.82
	OH		1.98 (s)

### Synthesis and characterization of beta-sitosterol-loaded chitosan nano-encapsulation

**Figure 3:**
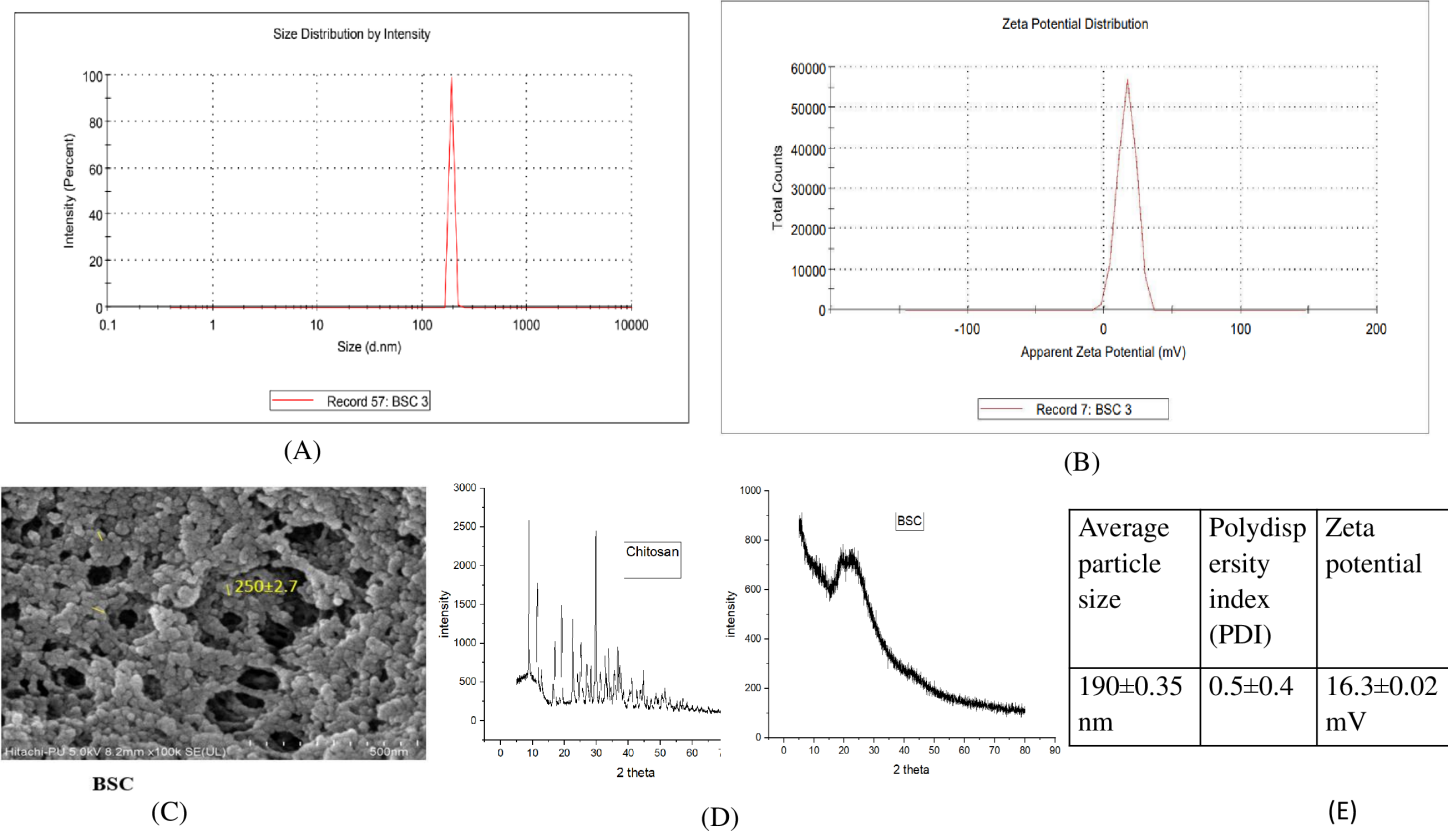
Characterization of chitosan-based nano beta-sitosterol (BSC). **(A, B)** Particle size distribution (190 ± 0.35 nm) and zeta potential distribution (16.3 ± 0.02 mV) spectrogram by DLS. **(C)** FE-SEM image represents spherical shape of chitosan-based β-sitosterol particles (BSC) and displays average size of 250 ± 2.7 nm (ImageJ software). **(D)** X-ray diffractogram of chitosan showing intense peak at 2θ values of 10° and 20°, while that of chitosan-based nano β-sitosterol (BSC) shows only light hump peak at 2θ value of 20°. **(E)** Particle size, polydispersity index (PDI), and zeta potential of nanoformulation. DLS, dynamic light scattering; FE-SEM, field emission scanning electron microscopy.

**Figure 4:**
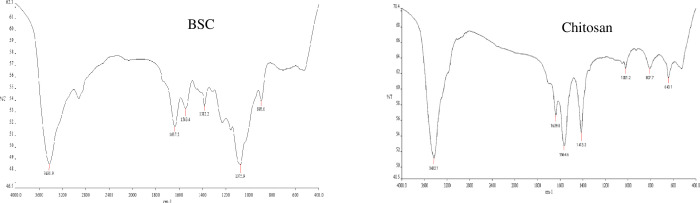
FTIR spectrogram of chitosan-based nano β-sitosterol (BSC) and chitosan. FTIR, Fourier transform infrared spectroscopy.

BSC was synthesized using the ionic gelation method with TPP as a crosslinker. As shown using the zeta sizer (Malvern), the average diameter, polydispersity index (PDI), and zeta potential distribution of nano beta-sitosterol were approximately 190 ± 0.35 nm, 0.592 ± 0.40, and 16.3 ± 0.02 mV, respectively (**Figures 3A, B, E**). Field emission scanning electron microscopy (FE-SEM) revealed that the spherical BSC nanoparticles had a diameter of 250 ± 2.7 nm, as analyzed using the ImageJ software (**Figure 3C**). XRD analysis was employed to investigate structural changes induced by β-sitosterol encapsulation within chitosan nanoparticles. Pure chitosan displayed prominent peaks at 2θ values of 10° and 20°, indicative of its crystalline nature. In contrast, the β-sitosterol-loaded chitosan nanoparticles exhibited a significantly reduced crystallinity, characterized by a broad peak around 20° (**Figure 3D**). FTIR analysis was employed to study β-sitosterol–chitosan interactions and to confirm successful loading. The IR spectra of chitosan and β-sitosterol encapsulate (BSC) are shown in **Figure 4**. The characteristic absorption bands of chitosan appeared at 1,639 and 1,564 cm^−1^, assigned to the C=O stretching and N–H bending vibrations of the amide I band, respectively. The absorption peak at 1,564 cm^−1^ disappeared, and a new absorption peak appeared at 1,543 cm^−1^, indicating that the phosphate group of TPP was connected to the amino site of chitosan. Peaks at 1,382 and 1,075 cm^−1^ denoted the isopropyl alcohol group and C–O bond of secondary alcohols, while the peak at 3,430 cm^−1^ denoted the extensive hydrogen bonding of chitosan and beta-sitosterol. The results demonstrate the successful encapsulation of β-sitosterol within the chitosan matrix using the ion crosslinking method, thereby achieving the primary objective of the study. Characterization data further validated the efficacy of this encapsulation technique.

### Encapsulation efficiency, loading capacity, and drug release profiles from polymeric nanoparticles

**Figure 5:**
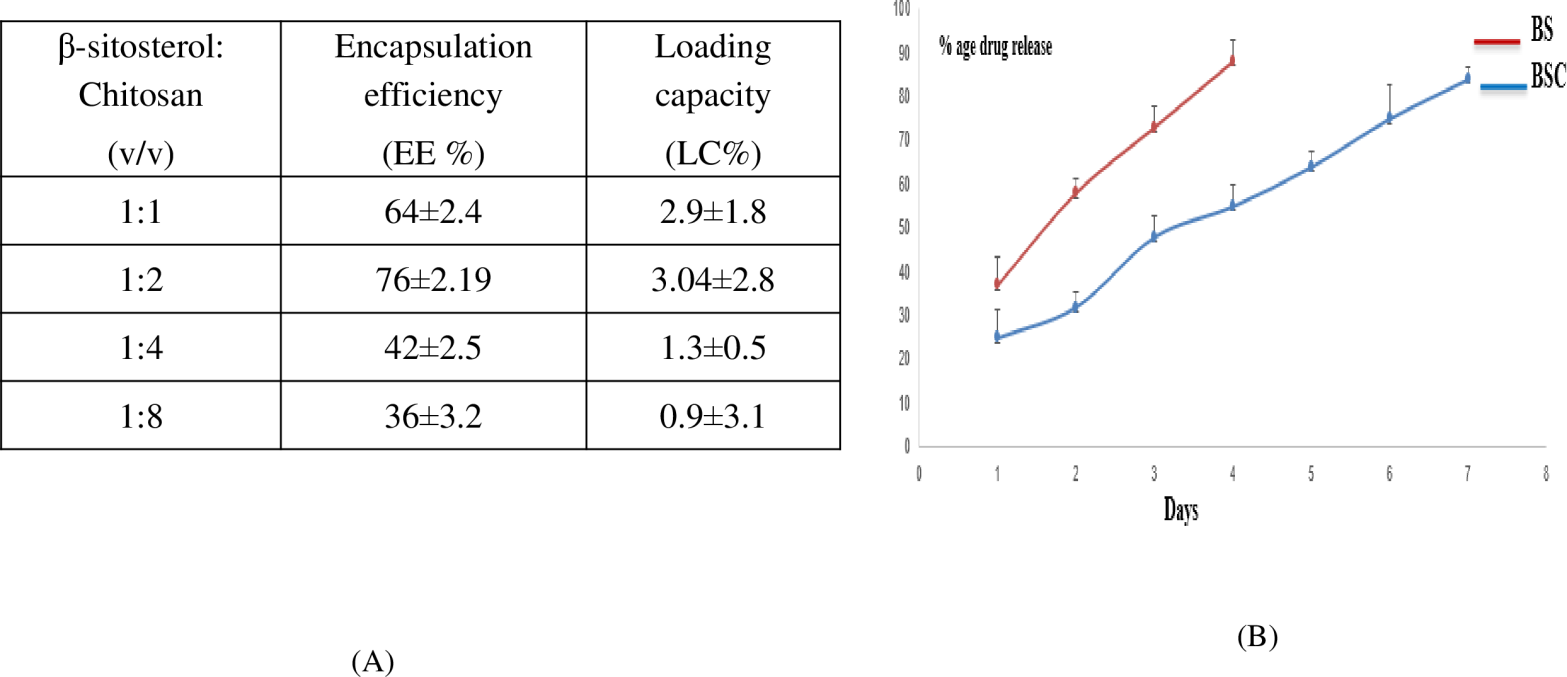
**(A)** Encapsulation efficiency (EE%) and loading capacity (LC%) with different ratios of beta-sitosterol and chitosan. **(B)***In vitro* drug release profile of nano beta-sitosterol (BSC) and beta-sitosterol (BSC) using dialysis bag. BS attained 90% relese by fourth day, while BSC continued to show sustained release beyond sixth day (mean ± SD, n = 3, p < 0.05).

Following the successful encapsulation of β-sitosterol within chitosan nanoparticles (BSC), the efficiency of the process using three key parameters, such as encapsulation efficiency (EE), loading capacity (LC), and *in vitro* drug release profiles, was evaluated. The maximum EE of BSC was found to be 76% ± 2.19% with a 1:2 v/v beta-sitosterol/chitosan formulation among the four different ratios of beta-sitosterol to chitosan (1:1, 1:2, 1:4, and 1:8) chosen for the study, with a loading capacity of 3.04% ± 2.8%. The other ratios showed lower EE; for example, 1:1 v/v β-sitosterol/chitosan showed EE of 64% ± 2.4%, 1:4 v/v β-sitosterol/chitosan showed EE of 42% ± 2.5%, and 1:8 v/v β-sitosterol/chitosan showed EE of 36% ± 3.2%. Regarding the LC parameter, the other ratios showed lower LC; for example, 1:1 v/v β-sitosterol/chitosan showed LC of 2.9% ± 1.8%, 1:4 v/v β-sitosterol/chitosan showed LC of 1.3% ± 0.5%, and 1:8 v/v β-sitosterol/chitosan showed LC of 0.9% ± 3.1% (**Figure 5A**). Furthermore, *in vitro* release studies using a dialysis bag method at pH 7 revealed significant differences between free BS and encapsulated beta-sitosterol (BSC) (**Figure 5B**). Free BS exhibited a burst release, with nearly 90% released within 4 days, potentially limiting its therapeutic effectiveness. In contrast, beta-sitosterol nanoform (BSC) displayed a sustained release profile, with continuous release of beta-sitosterol observed up to 7 days. The above results showed the slow and extended release of encapsulated molecules from the dialysis bag, acting like a semi-permeable membrane. The observed sustained release profile indicates the potential for prolonged delivery of beta-sitosterol compared to the free form.

### Anticancer potential of free beta-sitosterol and its nanoformulation

For establishing the anticancer potential of the molecule and its nanoformulation, several assays were conducted. Initially, *in vitro* cytotoxicity against various human cancer cell lines of different tissues, viz., colorectal (HCT-116), prostate (PC-3), lung (A549), and breast (MCF-7) cancers, was established. Among the tested cell lines, the highest *in vitro* cytotoxicity was observed against lung cancer cells (A549), which exhibited the greatest sensitivity to beta-sitosterol with an IC_50_ value of 11.93 ± 0.25 μg/mL. Other cancer cell lines showed varying sensitivities, such as HCT-116 with an IC_50_ value of 57.96 ± 0.4 μg/mL, PC-3 with an IC_50_ value of 42.3 ± 1.3 μg/mL, and MCF-7 with an IC_50_ value of 17.8 ± 0.7 μg/mL. However, relatively no significant inhibitory effect of beta-sitosterol was observed against the normal fr-2 breast cell line (IC_50_ > 100 μg/mL). Similarly, BSC exhibited IC_50_ values of 59.62 ± 0.36, 13.8 ± 0.26, 19.34 ± 1.2, and 43.87 ± 1.6 μg/mL against colorectal (HCT-116), lung (A549), breast (MCF-7), and prostate (PC-3) cancers, respectively. No significant inhibitory effect was observed against the normal (fr-2) breast cell line (IC_50_ value of >100 μg/mL). Notably, the IC_50_ values for BS and BSC displayed minimal variation.

### Clonogenic cell proliferation

**Figure 6:**
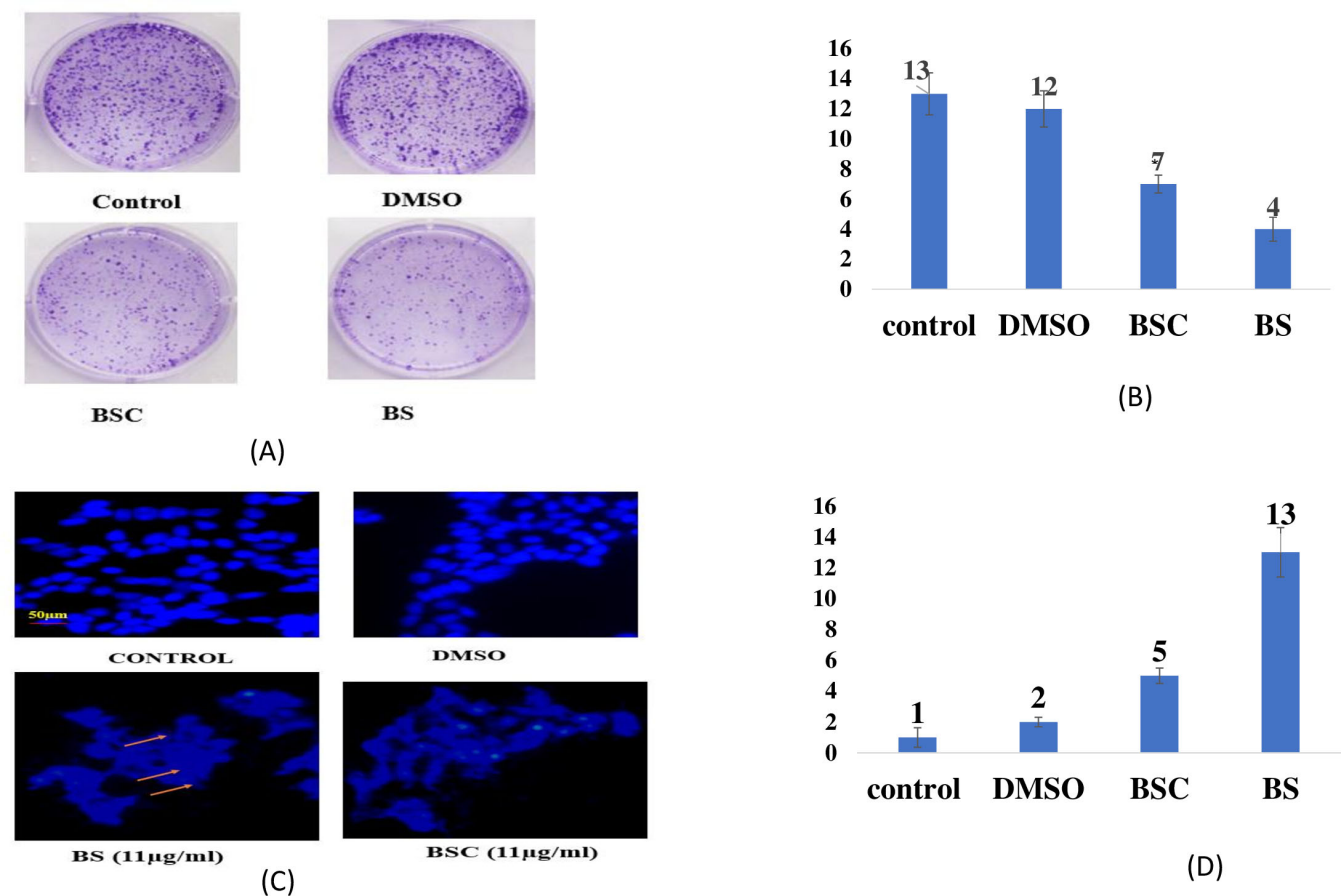
**(A)** Colony formation by the A549 cells after 72 hours of treatment with free β-sitosterol (BS) and chitosan-based nano β-sitosterol (BSC). **(B)** Graphical representation of average number of colonies per field analyzed at ×20 using fluorescence microscopy, control (13 ± 2.5 colonies), BSC (7 ± 2.1 colonies), and BS (4 ± 3.5). **(C)** Nuclear distortion exhibited by the A549 cells after 72 hours of treatment with free β-sitosterol (BS) and chitosan-based nano β-sitosterol (BSC), with elongated and enlarged nuclei represented by the arrows, and normal spherical nuclei of untreated A549 cells. **(D)** Graphical representation of average number of distorted nuclei per field analyzed at ×20 using fluorescence microscopy: BS (13 ± 1.5 distorted nuclei) and BSC (5 ± 1.3 distorted nuclei) (mean ± SD, n = 5, p < 0.05).

In order to determine the cell proliferation inhibition potential of BS and BSC, a colony formation assay was performed, wherein the number of colonies of A549 cells after 72 hours of molecule treatment was analyzed. Images were taken at ×20 using fluorescence microscopy, and the number of colonies per field was analyzed (no. of fields = 5). The results revealed a significant difference in A549 cell survival following treatment with BS and BSC at the same concentration (11 μg/mL). Notably, cells treated with BSC displayed a higher number of colonies per field (7 ± 2.1 colonies) compared to free BS treatment (4 ± 3.5 colonies), and untreated cells exhibited 13 ± 2.5 colonies per field (**Figures 6A, B**).

### Cellular nuclear distortion fluorescence imaging

Apoptosis measurement is often accomplished by fluorescence microscopy screening exemplary images of the apoptotic bodies and chromatin condensation resulting in nuclear distortion as compared to the healthy cells (28). This underlines the cellular processes that are characteristic of apoptosis. In biological systems, several microscopic techniques have been explored for characterization, wherein DAPI staining remains an important method of interest in nuclear staining because of its specificity and precise quantification ^12^. Therefore, in this study, fluorescence microscopy analysis using DAPI-stained cells was used to study the nuclear alterations and apoptotic body formation. A549 lung cancer cells were treated with both free BS and its nanoformulation (BSC) to assess induced nuclear alterations. Control A549 cells exhibited normal, rounded nuclei with homogeneous DAPI staining after 72 hours. In contrast, cells treated with BS and BSC displayed abnormal nuclear morphologies, including elongation, enlargement, and a bubble-like appearance. Here, the number of distorted nuclei at ×20 using fluorescence microscopy (no. of fields, n = 5) was analyzed. The results showed that when treated with the same concentrations (11 μg/mL), i.e., IC_50_ values of BS and BSC on lung (A549) cancer cell line (for 72 hours), a higher number of distorted nuclei per field was seen in the case of cells treated with free BS (13 ± 1.5 nuclei per field) with respect to cells treated with BSC (5 ± 1.3 nuclei per field), in comparison to the untreated cells that exhibited normal morphology (**Figures 6C, D**).

### Flow cytometric analysis of cell cycle progression

**Figure 7:**
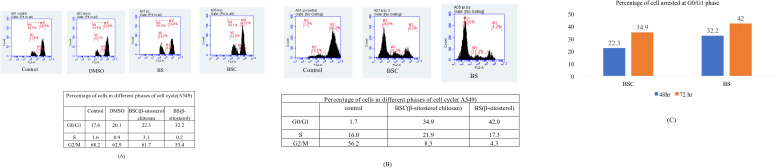
Cell cycle progression after 48 and 72 hours of treatment with β-sitosterol (BS) and chitosan-based nano β-sitosterol (BSC). **(A)** Flow cytometric determination of cell cycle progression after 48 hours of treatment of A549 cells with BS and BSC, depicting 32.2% of cells at G_0_/G_1_ phase in case of BS and 22.3% in case of BSC-treated A549 cells. **(B)** Flow cytometric determination of cell cycle progression after 72 hours of treatment of A549 cells with BS and BSC, depicting 42% of cells at G_0_/G_1_ phase in case of BS and 34.9% in case of BSC-treated A549 cells. **(C)** Graphical representation of comparative analysis of A549 cell cycle arrest at G_0_/G_1_ phase after 48 and 72 hours of treatment with BS and BSC (mean ± SD, n = 3, p < 0.05).

Since the results of the fluorescence microscopy demonstrated prominent nuclear distortion by the free molecule and its respective formulation against lung A549 cancer cells, the investigation was carried out to decipher the underlying mechanism of this growth inhibition in a time-dependent manner. Flow cytometric analysis was employed to measure and compare the effect of free molecule (BS) and its encapsulated formulation (BSC) on cell cycle progression by measuring the cellular DNA content in A549 cells. The results indicated that the treatment of lung A549 cancer cells with the IC_50_ values of BS and BSC resulted in a significant arrest at the G_0_/G_1_ phase in a time-dependent manner. The results showed that 22.3% of cells arrested at the G_0_/G_1_ phase by BSC-treated cells, as compared to 32.2% in the case of BS-treated cells, after 48 hours of treatment. After 72 hours of treatment, there were 34.9% of cells arrested at the G_0_/G_1_ phase in the case of BSC-treated cells, as compared to 42% in the case of BS-treated cells (**Figures 7A–C**).

### Detection of cellular apoptosis by flow cytometric analysis

**Figure 8:**
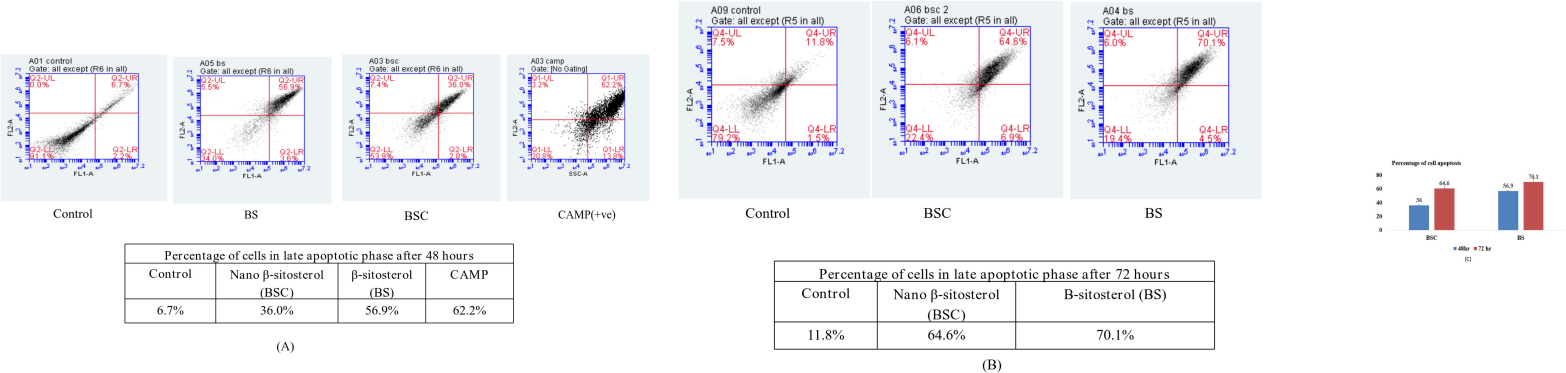
Detection of cellular apoptosis by flow cytometric analysis after 48 and 72 hours of A549 cells with same concentration (11 μg/mL) of free β-sitosterol **(B)** and chitosan-based nano β-sitosterol (BSC). **(A)** Flow cytometric determination of cell apoptosis after 48 hours of treatment of A549 cells with BS and BSC, depicting 60.2% of cells in late apoptotic phase in case of BS and 41.2% in case of BSC-treated A549 cells. **(B)** Flow cytometric determination of cell cycle progression after 72 hours of treatment of A549 cells with BS and BSC, depicting 71.4% of cells in late apoptotic phase in case of BS and 65.9% in late apoptotic phase in case of BSC-treated A549 cells. **(C)** Graphical representation of comparative analysis of A549 cell apoptosis after 48 and 72 hours of treatment with BS and BSC (mean ± SD, n = 3, p < 0.05).

To further confirm apoptosis, flow cytometric analysis was performed on Annexin V–Fluorescein isothiocyanate/Propidium iodide (FITC/PI)-stained A549 cells in a time-dependent manner. Annexin V–FITC, a cell membrane-impermeable dye, binds to the externalized phospholipid phosphatidylserine, a marker for early apoptotic cells, while propidium iodide binds to the DNA of late apoptotic cells (27). The abilities of cells to bind Annexin V–FITC/PI were measured using a flow cytometer, with the same concentration (11 μg/mL) of BS and BSC against A549 cells established. After 48 hours of treatment, there were 41.2% of cells in the apoptotic stage in the case of BSC-treated cells, as compared to 60.2% in the case of BS-treated cells. After 72 hours of treatment, there were 65.9% of cells in the apoptotic stage in the case of BSC-treated cells, as compared to 71.4% in the case of BS-treated cells. Camptothecin (CAMP) was used as a positive control for cell apoptosis, showing 66.3% of A549 cells in the late apoptotic stage (**Figures 8A–C**).

### *In silico* prediction of anticancer mechanism by molecular docking analysis

**Figure 9:**
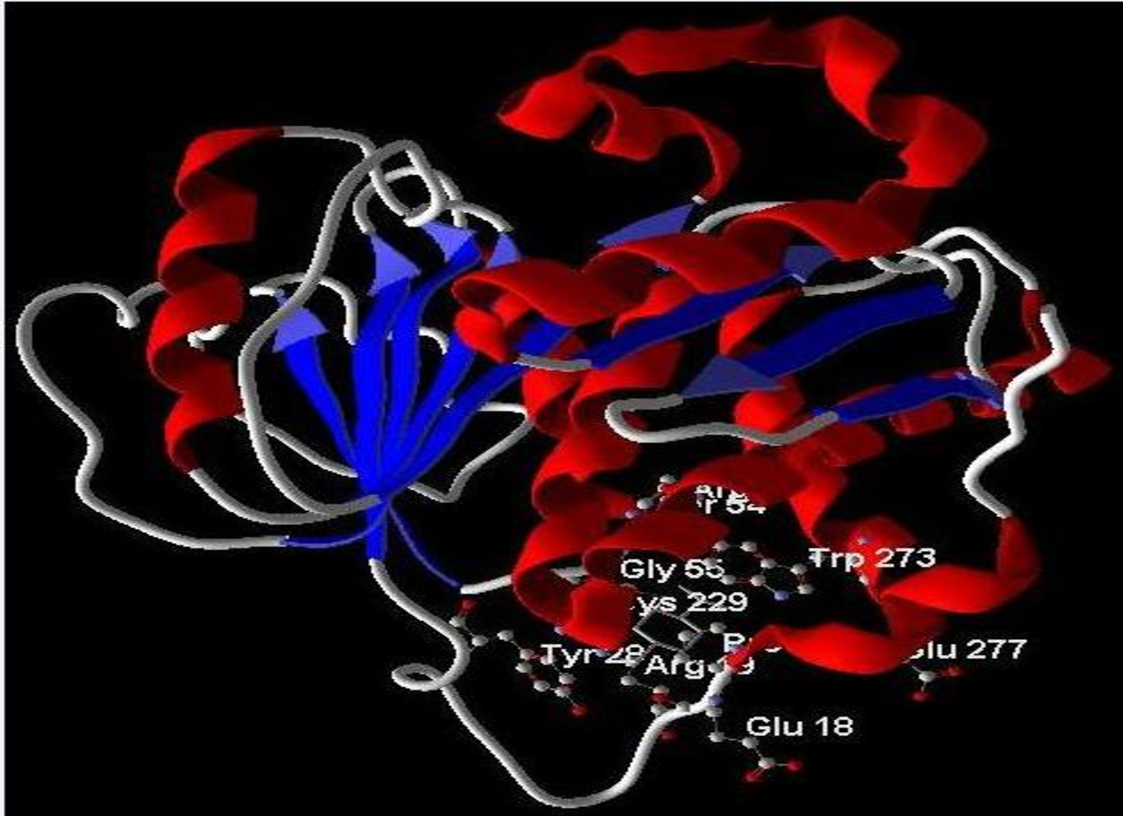
*In silico* docking of β-sitosterol against NRF1. NRF1, nuclear respiratory factor 1.

To elucidate the potential mechanism underlying the anticancer activity of beta-sitosterol against A549 cells, molecular docking analysis was performed targeting NRF1 protein, a transcription factor involved in cancer cell survival (19). The analysis revealed that beta-sitosterol exhibited the highest binding affinity for the NRF1 protein. Among the five docking scores obtained by positioning the ligand at different sites within the protein cavity, the highest docking score obtained was −927.14, with a re-rank score of −345.83 (**Table 2**) (**Figure 9**). The docking energy helped identify the optimal binding pose, and the most suitable target–ligand complex was ranked based on binding affinity and root mean square deviation (RMSD), which measures the average distance between atoms.

**Table 2:** Docking scores of β-sitosterol against NRF1 protein.

Name	Protein score	Ligand score	Molecular docking score	Re-rank score	RMSD	Docking score
[00]222284	222,284	1,537.77	−854.34	226.017	0	1,537.77
[01]222284	222,284	2,784.22	808.193	12,832.8	0	2,784.22
[02]222284	222,284	3,172.67	−875.86	468.314	0	3,172.67
[03]222284	222,284	4,515.44	1,834.93	24,763.2	0	4,515.44
[04]222284	**222,284**	**9,074.29**	**−927.14**	**−345.838**	**0**	**9,074.29**

NRF1, nuclear respiratory factor 1; RMSD, root mean square deviation. 04 ligand positioning site shows the highest molecular docking score.

## Discussion

Recent developments in plant-based drugs have shown significant potential in cancer chemotherapy, with approximately 70% of these compounds undergoing clinical trials at various stages (29). Therefore, this study focused on isolating anticancer molecules from *J. macrocephala* chloroform root extract, resulting in a potential subfraction (29JM). Using characterization techniques, subfraction 29JM was identified as beta-sitosterol with a yield of 0.6% (w/w). One report has claimed the isolation of β-sitosterol from *J. macrocephala* roots collected from the Western Himalayas without revealing its yield. However, β-sitosterol has been isolated from other shrubs like *Elaeagnus angustifolia* (0.5% w/w). β-Sitosterol’s unique structure, favorable properties, and low toxicity at higher doses make it a promising candidate for pharmaceutical development. However, its clinical application has been hindered by its poor bioavailability, which results from several factors, including low solubility, instability, limited tissue uptake and distribution, rapid clearance, and swift metabolism (30). To overcome these challenges, encapsulating β-sitosterol within nanoparticles offers a promising solution, significantly improving its bioavailability, enhancing drug accumulation at target sites, and enabling sustained release for more effective therapeutic outcomes. Natural biopolymers, particularly those used in nanocarrier formulations, are gaining attention as promising drug delivery systems (DDSs). In this study, chitosan, a natural biopolymer, was selected for the encapsulation of beta-sitosterol. Chitosan’s positive surface charge facilitates interaction with cell membranes, its high aqueous solubility enhances drug dispersion, and its bio-adhesive properties allow for prolonged retention, overall contributing to improved beta-sitosterol bioavailability (31, 32). Additionally, chitosan is widely accepted in various sectors of agriculture, food industries, and medical research owing to its US-FDA approval. In this study, β-sitosterol was encapsulated in chitosan through the ionic crosslinking method using TPP as an ionic crosslinker. This method used the electrostatic interactions between the amino-sugar of chitosan and the polyanions of TPP (33). A key advantage of this technique is its cost-effectiveness, while also avoiding the use of organic solvents, excessive mixing, or high temperatures, which are critical for preserving the bioactivity of the molecules in their native forms (31). The encapsulated β-sitosterol achieved a size of approximately 190 nm, falling well within the ideal range for passive tumor targeting, as many solid tumors have vascular pore cut-offs between 380 and 780 nm. Additionally, nanoparticles measuring 250 nm were considered optimal for passive targeting of tumors, given their compatibility with these vascular pore sizes (6). Zeta potential is a key physical property of particles in suspension, influenced by surface charge, and is critical for maintaining the stability of NPs in suspension. It is generally accepted that particles with zeta potentials greater than +30 mV or less than −30 mV exhibit sufficient electrostatic repulsion for colloidal stability (34). In this study, the zeta potential of the nano-encapsulation was recorded at 16.3 mV, indicating good stability, as the particles were adequately spaced to minimize aggregation. The positive surface charge of the nanoparticles also generates repulsive forces, further reducing the likelihood of aggregation in buffer solutions (35). Moreover, a PDI value of 0.592 reflects a more uniform particle size distribution. These findings, consistent with DLS results, suggest that the nanoparticle size is appropriate for potential applications in cancer targeting. The size complements strategies to address the issue of nanoparticle leakage outside tumors, rather than penetration (36). Their findings highlighted that nanoparticles smaller than 300 nm can effectively penetrate tumor cut-off pores. The XRD data from this study indicate a notable reduction in chitosan’s crystallinity, likely due to successful β-sitosterol incorporation and the formation of an amorphous structure, aligning with previous findings on β-sitosterol-loaded chitosan nanoparticles (37). A high loading efficiency was essential to achieve the desired therapeutic effect of the drug dose without compromising its biological activity. In this study, β-sitosterol was successfully loaded into the formulated nanoparticles with an efficiency of 76% using a chitosan concentration of 0.15% and 1:2 β-sitosterol:chitosan. β-Sitosterol is hydrophobic, whereas chitosan is hydrophilic and forms complexes through weak van der Waals and hydrophobic interactions. At a 1:2 ratio, adequate chitosan chains are accessible to encapsulate and stabilize the hydrophobic β-sitosterol molecules. Lower chitosan causes inadequate coating and poor dispersion of β-sitosterol due to its low aqueous solubility. High chitosan increases viscosity and deters proper nanoparticle formation. Therefore, a 1:2 ratio accomplishes an important stoichiometric and rheological balance, yielding nanoparticles with high encapsulation efficiency and better stability. These findings are consistent with those of Krishnamoorthy et al. (2021), who reported an encapsulation efficiency of 81.4% for 5 mg/mL beta-sitosterol in a 0.5% chitosan solution stirred for 12 hours. In contrast, the current protocol utilized 0.15% chitosan with continuous stirring for 4 hours. These findings emphasize that variations in nanoparticle preparation parameters, such as chitosan concentration and stirring time, can have a significant impact on encapsulation efficiency and loading capacity. For a drug delivery system to be effective, it must exhibit a high degree of drug association. The release from chitosan nanoparticles followed a typical biphasic pattern, with an initial rapid release phase followed by a slower, more controlled release phase (38). The results of this study demonstrated a sustained release profile of β-sitosterol from chitosan, highlighting its potential for prolonged delivery. A similar release pattern was reported where β-sitosterol encapsulated in chitosan showed sustained release over 48 hours at pH 7.2 (39). In our study, 90% of the release was achieved by day 4 at pH 7, whereas a 40% release within 48 hrs was previously observed (40). This variation may be due to differences in pH conditions between the two studies, although both show comparable overall release profiles. Following the successful encapsulation of β-sitosterol into chitosan nanoparticles (BSC), the anticancer potential of both pure BS and its nanoformulated counterpart was assessed. A series of experiments were conducted to compare the effects of free BS and BSC on lung cancer cells over time, with the aim of determining how encapsulation affects anticancer activity and whether it offers a more sustained therapeutic impact. The anticancer potential of β-sitosterol aligns with findings of β-sitosterol activity against A549 cells with an IC_50_ value of 20 μM (41). Similar cytotoxic activity against A549 cells with an IC_50_ value of 24 μM was reported by Rajavel et al. (2018), reinforcing β-sitosterol efficacy in cancer treatment. As a prevalent phytosterol, β-sitosterol has been implicated in inhibiting cancer growth through mechanisms such as cell cycle arrest, metastasis suppression, and apoptosis induction (42). Additionally, the mode of lung cancer cell death was investigated through a series of experiments, including colony formation assays, nuclear distortion assessments, apoptosis evaluations, and cell cycle analysis. These studies aimed to determine how encapsulation in chitosan nanoparticles affects the cytotoxic patterns over time, potentially validating the objective of sustained release. Cancer metastasis, driven by key cellular processes such as detachment, adhesion, migration, and invasion, is central to tumor progression (31, 43). The finding indicated that free β-sitosterol more effectively inhibited the colony-forming ability of A549 cells compared to its nano-encapsulated chitosan counterpart. This gradual release, potentially lasting beyond 72 hours, extends the presence of β-sitosterol in the cellular environment. These findings suggest that once internalized, the chitosan nanoparticles release β-sitosterol at a controlled pace, resulting in a sustained cytotoxic effect. This explains the observed difference in colony formation between free and nano-encapsulated β-sitosterol. Our findings align with those of Inamdar et al. (2018), who demonstrated that *in vitro* beta-sitosterol-loaded polymer nanoparticles exhibit delayed Tmax values, indicative of sustained beta-sitosterol release. Similarly, the fewer distorted nuclei observed in cells treated with BSC compared to free BS further supports the concept of slow, controlled release, potentially extending the efficacy of β-sitosterol over several days. This sustained efficacy highlights the advantage of chitosan nano-encapsulation, which facilitates controlled diffusion, where the nanoparticle’s polymer matrix acts as a barrier, moderating the drug’s release and contributing to the prolonged release profile, as demonstrated in previous studies (31). Various forms of programmed cell death are progressively involved in anticancer studies (44). To evaluate whether apoptosis and the inhibition of proliferation were linked through cell cycle arrest, we performed a cell cycle analysis using flow cytometry. Treatment with free BS induced a more pronounced cell cycle arrest at the G_0_/G_1_ phase, while BSC demonstrated a slower, sustained release over 48 to 72 hours. Both treatments significantly arrested the G_0_/G_1_ phase in a time-dependent manner at the same concentration, potentially inhibiting cancer cell proliferation by blocking cell cycle progression in lung cancer cells. Vundru et al. (2013) illustrated that Sitosterol treatment led to G_1_ arrest in human breast cancer MDA-MB-231 cells, corresponding to reduced levels of cyclin D1 and cyclin-dependent kinase (CDK) and increased levels of p21/Cip1 and p27/Kip1 proteins involved in inhibiting the kinase activity of CDK. Moreover, the lower percentage of viable cells in the late apoptotic phase observed with chitosan nano β-sitosterol compared to free β-sitosterol suggests a gradual release of the compound, which prolongs its availability within the cells and enhances its therapeutic effect. These findings are consistent with those of previous studies demonstrating the slow and controlled release of drugs from chitosan shells (45). The possible mechanism of osmosis can explain the slow release wherein the water from the surrounding environment, which has a lower drug concentration, flows into the chitosan shell where the concentration is higher, enabling a steady, zero-order release of the drug (46). The early apoptosis stage is marked by a round, cellular-shaped, dark cytoplasm following chromatin condensation, DNA fragmentation, and a subsequent decrease in size (39). Further apoptosis was observed via Annexin V/PI assay; percentages of apoptotic cells increased in 48 and 72 hours of treatment, as compared to the control. The treatments induced apoptosis in a time-dependent manner in A549 cells (47, 48). The lesser effect of encapsulated β-sitosterol versus its counterpart on A549 cells clearly indicates its slow release from the chitosan shell as compared to the burst release of the free molecule. Prolonged action translates to better therapeutic outcomes *in vivo* than *in vitro* due to the available tumor microenvironment provided by developing solid tumors. The *in vitro* cell culture systems cannot adequately absorb the formulations, while *in vivo* systems produce an enhanced permeability and retention (EPR) effect where chitosan macromolecule formulations preferentially accumulate in solid tumors due to highly permeable blood vessels and poor lymphatic drainage. *In silico* studies exploring the apoptotic potential of BS have centered on NRF1, a key cancer cell survival protein (49). In order to just give small mechanistic insight to our study, we selected NRF1 for an *in silico* study because earlier studies have shown that the NRF class of transcription factor upregulation in lung cancer supports tumor cell proliferation and rescues apoptosis, while its knockdown causes caspase-dependent cell death (50). These early findings suggest that targeting NRF1 could enhance the effectiveness of conventional cancer treatments, positioning it as a promising focus for future therapeutic strategies. Docking studies have shown that β-sitosterol effectively binds to NRF1, leading to its inactivation and disrupting its anti-apoptotic function, positioning β-sitosterol as a potential apoptosis-inducing agent. Collectively, these findings underscore β-sitosterol’s chitosan nanoformulation versatility and highlight its promising role, particularly in cancer therapy (51).

## Conclusion

This study isolated β-sitosterol from *J. macrocephala* Benth. roots, confirming its potential as a phytochemical source. The chitosan-based nanoformulation (BSC) achieved an encapsulation efficiency of 76% ± 2.19% and sustained release over 7 days, addressing β-sitosterol’s bioavailability challenges. Both free BS and BSC exhibited anticancer activity against A549 lung cancer cells (IC_50_: 11.93 ± 0.25 and 13.8 ± 0.26 μg/mL, respectively) with minimal toxicity to normal cells. BS induced stronger G_0_/G_1_ phase arrest and apoptosis, while BSC’s sustained release supports prolonged therapeutic effects. The identification of β-sitosterol’s binding to NRF1 (docking score, −927.38) provides insight into its anticancer mechanisms. By combining phytochemistry, nanotechnology, and oncology, this study offers a framework for advancing plant-derived cancer therapies.

## Data Availability

The original contributions presented in the study are included in the article/supplementary material. Further inquiries can be directed to the corresponding author.
